# Exploring the Impact of Initiating Endocrine Therapy on Metabolic Health in Early Breast Cancer: Protocol for the Prospective Follow-Up EMETA-Study

**DOI:** 10.2196/78589

**Published:** 2026-03-25

**Authors:** Lærke Nissen, Jonas Busk Holm, Signe Borgquist

**Affiliations:** 1 Department of Clinical Medicine, Aarhus University, Palle Juul-Jensens Boulevard 11, Aarhus N, 8200, Denmark, 45 91167294; 2 Department of Oncology, Aarhus University Hospital, Aarhus N, Denmark

**Keywords:** metabolic syndrome, breast neoplasms, antineoplastic agents, cardiometabolic risk factors, weight gain, hormonal

## Abstract

**Background:**

Adjuvant endocrine therapy is a cornerstone in managing estrogen receptor–positive early breast cancer but may adversely affect metabolic health, including weight gain, insulin resistance, and dyslipidemia. These changes increase the risk of cardiovascular disease and may influence breast cancer outcomes. However, the timing and magnitude of early metabolic changes following endocrine therapy initiation remain poorly characterized. Conventional definitions such as metabolic syndrome rely on dichotomous thresholds and may lack sensitivity to detect early treatment-related metabolic changes, highlighting the need for refined assessment approaches.

**Objective:**

This prospective follow-up study aims to investigate early metabolic effects of initiating adjuvant endocrine therapy in women with estrogen receptor–positive early breast cancer and to compare conventional and expanded approaches to metabolic health classification.

**Methods:**

This single-center, prospective observational study was conducted at Aarhus University Hospital, Denmark. Women aged≥18 years with early-stage estrogen receptor–positive breast cancer initiating adjuvant endocrine therapy and without pre-existing diabetes were eligible. Metabolic health was assessed at baseline and after 3 months using biometric measurements (weight, waist and hip circumference, waist-to-hip ratio, and blood pressure) and non-fasting blood samples (plasma glucose; hemoglobin A_1c,_ (HbA_1c_); lipid profile; and estradiol). The 3-month follow-up was selected to capture early metabolic changes while aligning with routine clinical care to minimize additional visits and reduce selection bias. Metabolic health will be evaluated using two conventional measures and two extended, exploratory measures. Conventional measures are metabolic syndrome (MetS), defined as meeting ≥3 of 5 established criteria (blood pressure ≥130/85 mmHg, triglycerides >2 mmol/l, high-density lipoprotein cholesterol <1.295 mmol/l, waist circumference >88 cm, and plasma glucose >7.8 mmol/l), and the Metabolic Syndrome z score (MetS-Z), a continuous standardized composite of the MetS components. Additional extended measures are exploratory: the extended MetS, which expands the standard MetS definition by incorporating low-density lipoprotein cholesterol (>3 mmol/l), body mass index (≥30 kg/m²), waist-to-hip ratio (>0.85), and HbA_1c_ (≥42 mmol/mol), and the EMETA score, a standardized composite of the extended MetS components calculated using the same approach as the MetS-*z* score.

**Results:**

The study was funded in July 2024. Recruitment occurred between November 2024 and April 2025, and follow-up was completed in September 2025. Statistical analyses are planned for February 2026, with results expected to be published in summer 2026.

**Conclusions:**

This study is expected to provide insights into early metabolic changes following initiation of adjuvant endocrine therapy and evaluate different approaches to classifying metabolic health. The aim to inform future research by helping to identify patients at increased risk of cardiometabolic complications and adverse breast cancer outcomes, warranting confirmation and validation of expanded metabolic measures in longer-term, larger cohorts.

## Introduction

Breast cancer is both the most frequent type of cancer and the leading cause of cancer-related mortality among women [1]. As survival rates improve, currently up to 90% in developed countries, there is increasing attention to the long-term health of breast cancer survivors [1,2]. Estrogen receptor-positive (ER+) early (stage I-III) breast cancer (EBC) represents the most common subtype, and adjuvant endocrine therapy (ET) is a cornerstone of its treatment [3,4]. While ET has significantly reduced recurrence and mortality, its metabolic side effects have emerged as a growing concern in survivorship care [4-6].

ET may influence metabolic health through estrogen deprivation [5,7]. Estrogen plays an important role in metabolic homeostasis by promoting insulin sensitivity, regulating lipid metabolism, and supporting vascular function [8,9]. By blocking estrogen receptors (tamoxifen) or suppressing aromatase activity (aromatase inhibitors, AI), ET may disrupt normal metabolic regulation, promoting weight gain, insulin resistance, and dyslipidemia [5,10-13]. These changes are risk factors for cardiovascular disease (CVD), which represents a major noncancer cause of morbidity and mortality among breast cancer survivors [14]. Furthermore, obesity and metabolic syndrome (MetS) at diagnosis have been associated with increased risk of breast cancer recurrence and mortality [15-18].

Although ET is typically administered for several years, routine clinical follow-up is often concentrated around treatment initiation. This limits systematic long-term monitoring of metabolic health in routine care, which highlights the importance of characterizing early metabolic changes. A short-term follow-up allows evaluation of initial changes in weight, lipid metabolism, and glycemic markers that may signal early metabolic vulnerability. A 3-month timeframe balances biological relevance with clinical feasibility, aligns with standard follow-up practice at the study site, and supports broad inclusion while minimizing selection bias.

Metabolic health is traditionally assessed using MetS, a dichotomous classification that identifies individuals as metabolically unhealthy when at least three of five criteria are met: hypertension (blood pressure ≥130/85), hypertriglyceridemia (triglycerides >2 mmol/l), low HDL (HDL <1.295 mmol/l), central obesity (waist circumference >88 cm), or hyperglycemia (plasma glucose >7.8 mmol/l) [19]. However, this binary approach may overlook subtle or early metabolic changes that do not meet clinical thresholds. [19,20]. An established alternative is the Metabolic Syndrome *z* score (MetS-*z* score), a continuous composite measure of standardized MetS components and widely used in research to assess metabolic health along a continuum [21-24]. However, both MetS and MetS-Z omit several cardiometabolic indicators relevant to breast cancer survivorship, including low-density lipoprotein (LDL), hemoglobin A_1c_ (HbA_1c_), BMI, and waist-to-hip ratio (WHR) [13,25,26]. To address this limitation, this study introduces two expanded measures of metabolic health, for exploratory and hypothesis-generating purposes: An extended MetS definition and the EMETA score, a dichotomous classification and a composite *z* score using the same approach as the MetS-*z* score, both incorporating the extended metabolic variables.

The aim of this study is to evaluate the early impact of initiating adjuvant endocrine therapy on metabolic health in women with ER+ EBC and to compare conventional and expanded approaches to classify metabolic health.

## Methods

### Study Population

This prospective, single-center observational study enrolled 112 patients with ER+ EBC who initiated adjuvant ET. Over a six-month period from November 2024, participants were recruited from the Department of Oncology, Aarhus University Hospital (AUH), Denmark. Patients were invited to participate during the consultation ET was prescribed. Participants received usual care throughout the study.

### Eligibility Criteria

Patients were eligible if they met the following criteria: age ≥18 years, diagnosis of invasive ER+ EBC, and initiation of adjuvant ET. Up to three days of ET treatment were allowed, to allow for the timeframe of informed consent.

Patients were excluded for enrollment in case of pregnancy or lactation, pre-existing diabetes (type I or II), more than three dispenses of ET, or any psychological, familial, sociological, or geographical conditions potentially hampering compliance with the study protocol and follow-up schedule. These conditions were discussed with the patient before trial registration. Patients with pre-existing diabetes were excluded because their chronic metabolic dysregulation and glucose-lowering treatments could confound assessment of early metabolic changes. Patients were excluded if metastatic breast cancer was diagnosed in the period between study inclusion and the 3-month follow-up. As the study did not involve any intervention administered by clinical staff beyond routine care, there were no additional eligibility criteria for sites or individuals delivering interventions.

### Study Design

**Figure 1.**
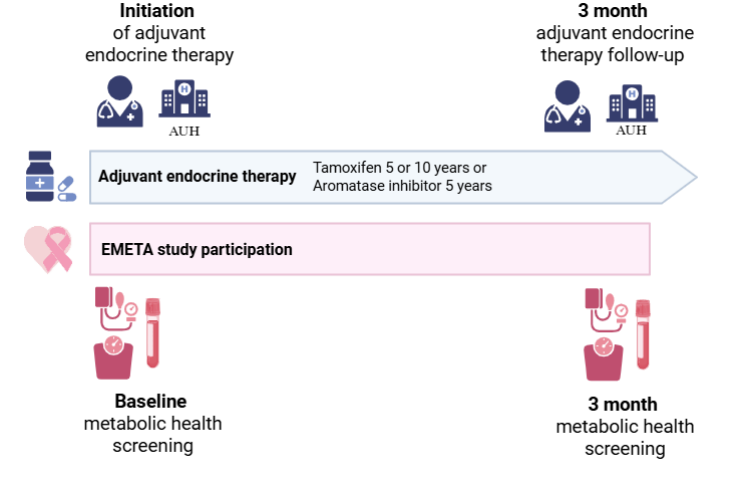
EMETA study design and participation created in BioRender [27] is licensed under CC BY 4.0 [28]. AUH: Aarhus University Hospital.

Participants underwent two metabolic health screenings: one at baseline and one at 3-month follow-up (Figure 1). The baseline screening was performed on the same day as study inclusion. The 3-month follow-up screening was performed on the same day as the 3-month ET follow-up. This design minimized additional study-related visits and supported participant retention.

### Exposure

The exposure of interest was initiation of ET over a 3-month period. ET consisted of either tamoxifen (20 mg daily) or an AI (letrozole 2.5 mg daily or exemestane 25 mg daily), prescribed according to standard clinical practice.

### Outcomes

The primary outcomes were selected to address whether initiation of ET is associated with early changes in overall metabolic health. Given the short-term follow-up and the aim to capture early, potentially subtle metabolic alterations, the primary outcomes include an anthropometric and a composite metabolic measure.

The primary outcomes are:

1. Change in mean BMI from baseline to 3-month follow-up
2. Change in mean MetS-Z from baseline to 3-month follow-up

BMI was chosen because weight changes are a well-known sideeffect of ET and may occur early following ET initiation, representing a clinically meaningful marker of metabolic health. Although short-term changes may be modest, even small increases may indicate emerging metabolic vulnerability. As a continuous composite measure, the widely used MetS-*z* score was selected to capture multidimensional metabolic changes that may not be reflected by individual metabolic parameters alone [21-24,29].

Secondary outcomes were selected to further characterize early metabolic changes and to compare expanded and conventional approaches to metabolic health classification.

The secondary outcomes include:

1. Change in the prevalence of MetS from baseline to 3-month follow-up
2. Comparison of the prevalence of MetS and extended MetS at 3-month follow-up
3. Change in mean EMETA score from baseline to 3-month follow-up
4. Change in mean values of individual metabolic parameters from baseline to 3-month follow-up, including waist circumference, hip circumference, WHR, blood pressure, and circulating levels of HbA_1c_, total cholesterol, triglycerides, LDL cholesterol, HDL cholesterol, and estradiol

The extended MetS and EMETA score are included as exploratory outcomes to assess whether broader composite measures capture early metabolic changes not identified by conventional definitions. Analysis of individual metabolic parameters provides insight into which metabolic domains are most affected shortly after ET initiation.

### Measurements

The screening procedures included biometric measurements and blood sample collections. The biometric measurements were height, weight, hip and waist circumference, and blood pressure. Non-fasting venous blood samples were analyzed for HbA_1c_, plasma glucose, hemoglobin, total cholesterol, triglycerides, LDL, HDL, and circulating estradiol.

### Biometric Measurements

The biometric measurements were measured through the following protocols. Weight was measured using a standardized scale with participants wearing light clothing and no shoes. Height was recorded with participants standing straight against a wall-mounted measuring tape without shoes. Waist circumference was measured 5 cm above the center of the navel and hip circumference at the widest part of the hip, both using a stretch-resistant tape, ensuring the tape was parallel to the floor and snug but not compressing the skin. Blood pressure was measured using a calibrated cuff on the upper arm after participants had rested for 5 minutes, with three consecutive readings taken with the participant seated both feet on the floor and both arms on their legs.

### Blood Samples

Nonfasting venous blood samples were analyzed by the Department of Clinical Biochemistry, AUH for levels of HbA_1c_, glucose, total cholesterol, triglycerides, LDL, HDL, estradiol, and sex-hormone binding globulin. The blood samples were destroyed immediately after analysis. Results outside standard values were reviewed by consultant physicians and patients, and their general practitioners were notified with follow-up recommendations when necessary.

### Data Collection and Management

Information on patient demographics, treatment modalities, and cancer-specific characteristics were collected through a manual search of electronic medical journals, including pathological records. All data were securely stored in a RedCap database hosted by Aarhus University.

To promote participant retention and ensure complete follow-up, the 3-month appointments were booked by hospital secretaries as part of routine care. If a participant was not scheduled for a follow-up appointment, trial personnel proactively contacted the participant to arrange the study visit. If participants discontinued ET, outcome data were still be collected at the 3-month follow-up visit, unless consent was withdrawn.

### Cohort Size

The study aimed to include 112 participants over the six-month enrollment period. The target was based on an annual referral rate of approximately 360 patients to the Department of Oncology, AUH, for adjuvant systemic therapy, of which approximately 90% were eligible for ET due to ER+ EBC, and assuming a 70% participation rate among eligible patients.

Statistical power calculations supported the sample size. Based on a comparable study with a similar population [30], where the standard deviation of the change in BMI over 3‐5 years after diagnosis was 3, a cohort of 112 participants provides >80% power to detect a true change in BMI of 0.8 kg/m^2^ at a 5% significance level. Furthermore, other studies have found larger BMI changes over shorter periods. For instance, Heideman et al observed a mean BMI change of 2.0 kg/m^2^ (± 4.9) after one year, with 26% of patients gaining ≥5 kg following adjuvant treatment [31].

### Statistical Analysis

#### Definition of Analytic Values

BMI will be calculated as weight (kg)/height^2^ (m). WHR will be calculated as waist circumference divided by hip circumference. Blood pressure will be calculated as the average of three measurements. Middle arterial pressure will be calculated as 2 times diastolic blood pressure added systolic blood pressure divided by 3.

**Figure 2.**
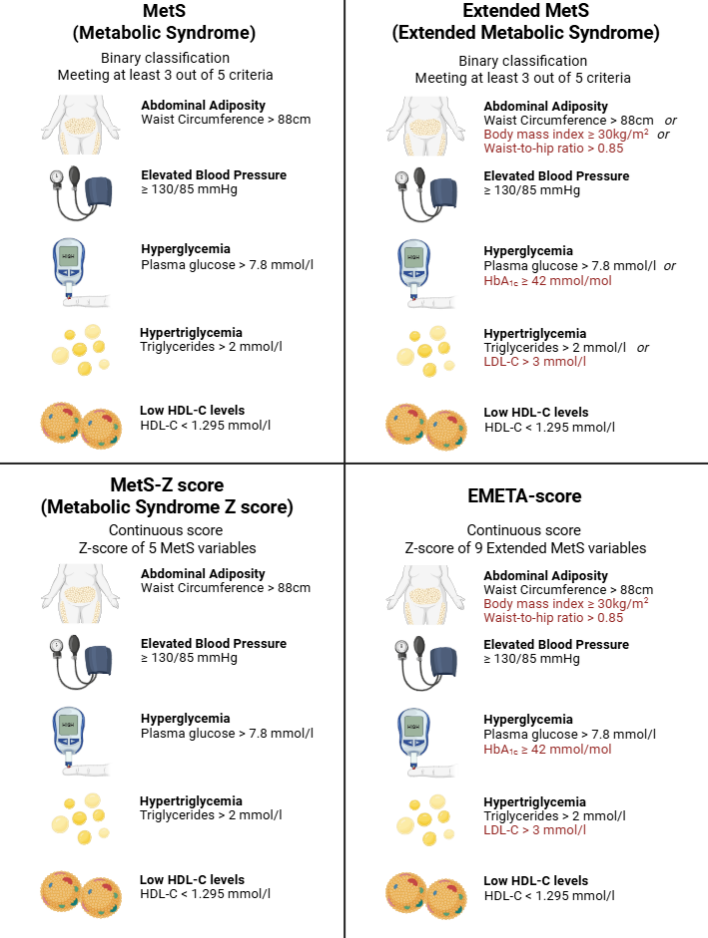
Comparison of MetS, extended MetS, MetS-Z, and EMETA scores. Red text: Variables specific to the extended MetS and EMETA score. HDL: High-density lipoprotein, LDL: Low-density lipoprotein. HbA_1c_: Hemoglobin A_1c_. Created in BioRender. Busk Holm, J. (2025) https://BioRender.com/

MetS will be defined as meeting at least three of five criteria: hypertension (blood pressure ≥130/85), hypertriglyceridemia (triglycerides >2 mmol/l), low HDL (HDL <1.295 mmol/l), central obesity (waist circumference >88 cm), or hyperglycemia (plasma glucose >7.8 mmol/l) (Figure 2).

Extended MetS will be defined as meeting at least 3 of the five criteria: hypertension (blood pressure ≥130/85), hypertriglycemia (triglycerides >2 mmol/l or LDL >3 mmol/l), low HDL (HDL <1.295 mmol/l), adiposity (waist circumference >88 cm, BMI ≥30, or WHR >0.85), or hyperglycemia (plasma glucose >7.8 mmol/l or HbA_1c_ ≥42 mmol/mol).

#### *z* Score Calculations

The MetS-*z* score will be calculated as a standardized composite score of the five MetS components. Standardization will be achieved by subtracting ATP III criteria from each individual’s value of waist circumference (WC), triglycerides (TG), middle arterial pressure (MAP), plasma glucose (PG), and inverted HDL, dividing by the baseline cohort standard deviation (σ).


(1) $$MetS\text{-}Z=\frac{WC-88}{\sigma_{wc}}+\frac{TG-2}{\sigma_{TG}}+\frac{MAP-100}{\sigma_{MAP}}+\frac{PG-7.8}{\sigma_{PG}}-\frac{HDL-1.295}{\sigma_{HDL}}$$

The EMETA score is a exploratory composite *z* score that will be calculated using the same approach as MetS-*z* score, based on the extended MetS components.


(2) $$\begin{array}{rl} \text{EMETA}= & \frac{WC-88}{\sigma_{WC}}+\frac{BMI-30}{\sigma_{BMI}}+\frac{WHR-0.85}{\sigma_{WHR}}+\frac{TG-2}{\sigma_{TG}}\\ -\frac{HDL-1.295}{\sigma_{HDL}}+\frac{LDL-3}{\sigma_{LDL}}+\frac{MAP-100}{\sigma_{MAP}}+\frac{PG-7.8}{\sigma_{PG}}+\frac{HbA1c-42}{\sigma_{HbA1c}} \end{array}$$

#### Covariables

Information on the following co-variables has been collected from medical records and will be included in adjusted analyses. These include patient demographics: age, weight, height, Carlson Comorbidity score (0, 1‐2, 3‐4, ≥5), current comorbidities (none, cardiopulmonary disease, neurological, dyslipidemia, musculoskeletal, other cancers, abdominal, urogenital, endocrine, dermatological, others), smoking status (never smoker, former smoker, active smoker), civil status (living alone, living with a partner), and area of living (rural/city). Cancer-specific characteristics: date of breast cancer diagnosis, disease stage (T+N), human epidermal growth factor receptor 2 status, histological type, grade of malignancy, and previous breast cancer history. Treatment modalities included duration and type of neo-adjuvant and adjuvant therapy (including supportive prednisolone treatment), date of breast cancer surgery, and radiotherapy.

### Analytical Approach

Statistical analyses will be conducted using Stata (version 18.5), with a 5% significance level for hypothesis testing. Changes in continuous primary outcomes (BMI and MetS-*z* score) will be analyzed using linear regression or linear mixed-effects models, as appropriate, to estimate mean within-person changes from baseline to 3-month follow-up. Changes in secondary continuous outcomes, including the EMETA score and individual metabolic parameters, will be analyzed using similar regression-based approaches. Changes in categorical outcomes (MetS and Extended MetS prevalence) will be analyzed using logistic regression models.

To account for exposure heterogeneity and treatment-related confounding, type of ET (tamoxifen vs aromatase inhibitor) and prior cancer treatments with potential metabolic impact, including neoadjuvant or adjuvant chemotherapy and supportive corticosteroid use, will be included as covariates in all adjusted analyses.

To address the secondary research question of whether certain patients are more vulnerable to early metabolic health deterioration following the initiation of ET, exploratory subgroup analyses will be conducted across both primary and secondary outcomes. These analyses will examine whether metabolic changes differ according to baseline and treatment-related characteristics, including age, BMI category, baseline metabolic status, menopausal status, type of ET, prior chemotherapy, smoking status, and comorbidity score. These subgroup analyses will be considered hypothesis-generating. Missing data will be assessed for randomness and extent. If data are missing at random, appropriate methods such as multiple imputation or mixed-effects models will be applied. Sensitivity analyses will be conducted to evaluate the potential impact of missing data on the study’s primary and secondary outcomes.

### Ethical Considerations

This study was conducted in agreement with the Declaration of Helsinki and the laws and regulations of Denmark, whichever provided the highest level of patient protection. The study protocol was approved by the Scientific Ethics Committee for the Central Denmark Region (case nr: 1-10-72-122-24). Any important modifications to the protocol (eg, changes to eligibility criteria, outcomes, or analysis methods) were communicated to the relevant ethics committee and trial registry (ClinicalTrials.gov ID NCT06623903).

#### Informed Consent

Written informed consent was obtained from all participants before study entry, and any study-specific procedures were performed. This process was conducted in accordance with national and local regulatory requirements. Each consent form had to be signed and personally dated by the participant.

#### Risks and Patient Inconvenience

The risk of adverse events associated with study participation was minimal. Rarely, minor bleeding or inflammation could occur at the blood sample puncture site. No long-term adverse events were anticipated. Participation required two additional blood samples and an approximate extension of 30 minutes during two oncology department visits (at study inclusion and the three-mo follow-up). Participants were covered under the standard patient compensation system in Denmark for any unexpected harm related to study procedures.

#### Data Protection and Privacy

Personal information collected during the study was securely stored in the RedCap database hosted at Aarhus University. Data were pseudonymized to enable linkage with clinical records, with access to identifiable information restricted to authorized study personnel. All data were stored on secure, password-protected servers with two-factor authentication. All data were processed in accordance with the Danish Data Protection Act and the European General Data Protection Regulation (GDPR). The project was registered in the internal directory of the Central Denmark Region.

#### Dissemination of Results

Study findings will be disseminated in international peer-reviewed journals and shared at local journal clubs, scientific meetings, and at least one international congress. Study participants who consented at study inclusion to receive study results will be provided with a lay summary of the findings, supporting transparency and engagement in survivorship care. Patients or members of the public were not involved in the design, conduct, or reporting of this study.

#### Monitoring

A formal Data Monitoring Committee was not established for this study, as this was a noninterventional, observational trial involving minimal risk. Instead, the study was overseen by the study personnel (the authors) who were responsible for the daily conduct and integrity of the study. The study personnel completed and monitored all data collection to ensure protocol adherence, data quality, and participant safety. As the study sponsor (AUH) did not influence design, data analysis, or reporting, oversight by the study team was considered both appropriate and independent in practice. No interim analyses or formal stopping guidelines were planned, given the short follow-up period and non-interventional design. All data will be analyzed following the completion of the follow-up period.

## Results

The study was funded in July 2024. Participant recruitment occurred from November 2024 through April 2025, during which 112 of 166 eligible patients were enrolled. Among the 54 eligible patients who were not enrolled, 18 declined participation, 26 were not informed of the study by their treating practitioner, and 10 elected not to initiate adjuvant ET. All enrolled participants completed baseline assessments, and follow-up data collection was completed in September 2025. No deviations from the approved study protocol occurred, and no protocol amendments were made following ethical approval. Data cleaning and statistical analyses are scheduled to commence in February 2026, with dissemination of the final study results anticipated in July 2026.

## Discussion

This study is anticipated to indicate that initiation of ET in women with ER+ EBC will be associated with modest early metabolic changes over the first three months of treatment. These changes may include small increases in BMI and selected lipid-related parameters, while markers with longer biological response times, such as HbA_1c_, may show minimal or no short-term change. Extended and continuous measures of metabolic health may capture early alterations not identified by conventional MetS definitions. Importantly, these analyses are exploratory and intended to evaluate sensitivity and feasibility rather than to establish clinical thresholds or predictive value.

Previous studies have linked ET with weight gain, insulin resistance, and dyslipidemia, all of which increase cardiovascular risk and may impact breast cancer outcomes [5,10-12,14,32]. However, most prior work has focused on longer-term outcomes or relied on dichotomous classifications. By evaluating both conventional and expanded measures within a short-term framework, this study contributes to understanding how early metabolic changes may be characterized following ET initiation.

Key strengths of this study include its prospective design with paired baseline and 3-month follow-up data, allowing within-subject comparison of early metabolic changes. The use of both conventional and expanded metabolic measures enables comparison of different classification approaches and addresses known limitations of binary definitions. The short follow-up period and alignment with routine clinical care minimize additional participant burden, supporting broad inclusion and reducing the risk of selection bias related to study participation.

The study is conducted at a single center, which may limit generalizability beyond similar healthcare settings. Exclusion of patients with pre-existing diabetes, while necessary to reduce baseline metabolic confounding, restricts applicability to a population that commonly receives endocrine therapy. In addition, although efforts were made to minimize attrition, informative dropout cannot be excluded, particularly if early metabolic or treatment-related symptoms influenced follow-up participation. The modest sample size limits power for subgroup analyses, which are therefore considered exploratory.

Overall, this study aims to inform future research and clinical practice by clarifying early metabolic patterns following ET initiation and by evaluating alternative approaches to metabolic health assessment. Larger and longer-term studies are needed to determine whether early metabolic changes are associated with subsequent cardiovascular risk or breast cancer outcomes, and to validate expanded metabolic measures before any potential clinical application.

## Abbreviations

AI: aromatase inhibitor
AUH: Aarhus University Hospital
CVD: cardiovascular disease
EBC: early breast cancer
ER+: estrogen receptor positive
ET: adjuvant endocrine therapy
HbA_1c_: hemoglobin A_1c_
HDL: high-density lipoprotein
LDL: low-density lipoprotein
MetS: metabolic syndrome
MetS-Z score: Metabolic Syndrome z score
WHR: waist-to-hip ratio
